# The articulation of integration of clinical and basic sciences in concept maps: differences between experienced and resident groups

**DOI:** 10.1007/s10459-015-9657-2

**Published:** 2015-12-21

**Authors:** Sylvia Vink, Jan van Tartwijk, Nico Verloop, Manon Gosselink, Erik Driessen, Jan Bolk

**Affiliations:** ICLON, Leiden University, Wassenaarseweg 62A, 2333 AL Leiden, The Netherlands; Centre for Teaching and Learning, Educational Development and Training, Faculty of Social and Behavioural Sciences, Utrecht University, PO Box 80.140, 3508 TC Utrecht, The Netherlands; PSYVEL, Straatweg 94, 3051 BL Rotterdam, The Netherlands; Department of Education Development and Research, Maastricht University, Tongersestraat 53, 6211 LM Maastricht, The Netherlands; Leiden University Medical Centre, PO Box 9600, 2300 RC Leiden, The Netherlands

**Keywords:** Concept mapping, Curriculum development, Teacher learning, Integration of clinical and basic sciences, Expertise differences, Knowledge elicitation

## Abstract

To determine the content of integrated curricula, clinical concepts and the underlying basic science concepts need to be made explicit. Preconstructed concept maps are recommended for this purpose. They are mainly constructed by experts. However, concept maps constructed by residents are hypothesized to be less complex, to reveal more tacit basic science concepts and these basic science concepts are expected to be used for the organization of the maps. These hypotheses are derived from studies about knowledge development of individuals. However, integrated curricula require a high degree of cooperation between clinicians and basic scientists. This study examined whether there are consistent variations regarding the articulation of integration when groups of experienced clinicians and basic scientists and groups of residents and basic scientists-in-training construct concept maps. Seven groups of three clinicians and basic scientists on experienced level and seven such groups on resident level constructed concept maps illuminating clinical problems. They were guided by instructions that focused them on articulation of integration. The concept maps were analysed by features that described integration. Descriptive statistics showed consistent variations between the two expertise levels. The concept maps of the resident groups exceeded those of the experienced groups in articulated integration. First, they used significantly more links between clinical and basic science concepts. Second, these links connected basic science concepts with a greater variety of clinical concepts than the experienced groups. Third, although residents did not use significantly more basic science concepts, they used them significantly more frequent to organize the clinical concepts. The conclusion was drawn that not all hypotheses could be confirmed and that the resident concept maps were more elaborate than expected. This article discusses the implications for the role that residents and basic scientists-in-training might play in the construction of preconstructed concept maps and the development of integrated curricula.

## Introduction

During their training medical students need to learn to connect their clinical and basic science knowledge in order to become proficient doctors (Boshuizen and Schmidt 2000). Medical curricula aim to scaffold this learning (Dahle et al. 2002; Kulasegaram et al. 2013). Making integration of clinical and basic sciences explicit in concept maps enables us to determine the content of an integrated curriculum (Weiss and Levison 2000) and can support student learning (Cutrer et al. 2011; Kinchin et al. 2008; Rendas et al. 2006). Concept mapping helps teachers to achieve consensus about which concepts and interrelations to adopt in an educational programme, and in what order (Harden 2001). Concept maps constructed by teachers (hereafter referred to as ‘preconstructed concept maps’) turn out to support student learning: they serve as a road map during the acquisition of new knowledge (Cutrer et al. 2011; Nesbit and Adesope 2006; O’Donnell et al. 2002). Concept maps articulate concepts and their interrelations in a hierarchical way (Novak 2002) and might thus visualize the relations between clinical and basic science concepts that should be understood in order to understand a medical subject (Kinchin et al. 2008; Weiss and Levison 2000). It is for this reason that they are put forward as promising instruments for the articulation of integration (Daley and Torre 2010; Kinchin et al. 2008; Weiss & Levison 2000). There are not many concept mapping studies focusing on the learning effects on students’ integrated use of clinical and basic science knowledge (Cutrer et al. 2011; Gonzalez et al. 2008). However, there are studies demonstrating general learning effects when students’ learning is guided by concept maps that are constructed by teachers (Cutrer et al. 2011; Rendas et al. 2006). Studies about the use of other preconstructed schematizations also point to learning benefits for students (Blissett et al. 2012).

### Constructors of preconstructed concept maps

Using preconstructed concept maps intended to articulate integration of clinical and basic sciences in medical education raises the question of who would be the preferred constructors. Most authors take the position that for medical programmes preconstructed concept maps and other preconstructed schematization formats should preferably be constructed by experienced clinicians (Blissett et al. 2012; Cutrer et al. 2011; Edmondson 1994). However, preconstructed concept maps that are constructed by experienced clinicians might not be optimal for educational purposes. They might be too complex because the number of concepts, links and layers in hierarchies (Novak 2002), increases with the expertise of its constructor (Edmondson and Smith 1998; Rendas et al. 2006) and consequently might impose a task on students that is too far above their current knowledge level. It is then unlikely that learning will take place (Kirschner et al. 2006).

Neither do we know how and to what extent constructors of different expertise levels articulate integration of clinical and basic sciences in concept maps, nor whether their concept maps differ consistently with respect to articulation of integration of clinical and basic sciences. What we do know is that experienced clinicians and residents both possess theoretical and practical medical knowledge but differ in the way that clinical and basic science knowledge are embedded in their knowledge base (Boshuizen and Schmidt 2000). This difference might be visualized in their concept maps because they visualize the constructors’ understanding (Kassab and Hussain 2010; Rendas et al. 2006; West et al. 2000).

Integration in medical curricula is primarily a joint effort of clinicians and basic scientists (Harden 2000). Studies on curricula that have been successful in improving integration of clinical and basic sciences report intense cooperation of clinicians and basic scientists (Dahle et al. 2002; Wilkerson et al. 2009). For concept maps intended to structure course content or to guide student learning in an integrated curriculum, this implies that they should preferably be constructed by *groups* of clinicians and basic scientists, in order to achieve consensus about the clinical and basic science concepts, and particularly their interrelations, that are considered relevant to teach. Moreover, the information gap between constructors stimulates the elicitation of their joint knowledge (Novak 2002). Therefore, if we aim to articulate integration in concept maps intended for curriculum design and student learning, we should know more about the extent to which groups of constructors of different expertise levels articulate integration in their concept maps.

### Integration of clinical and basic sciences in concept maps

Concept mapping is put forward as a knowledge elicitation technique (Hoffman and Lintern 2006; Kinchin et al. 2008) and is used to visualize individual knowledge improvement (Rendas et al. 2006; West et al. 2002). Consistent differences between groups of experienced clinicians and groups of less experienced clinicians and basic scientists have not yet been investigated. Expectations about these differences can only be fuelled by literature on the knowledge base of individual clinicians (Boshuizen and Schmidt 2000; Eva et al. 2002a; Van de Wiel et al. 2000). There appear to be salient differences between experienced clinicians and residents with respect to the organization of their knowledge base and the way that clinical and basic science knowledge is related. Such insights enable us to hypothesize differences between groups of experienced clinicians and basic scientists on the one hand and less experienced groups on the other hand if their knowledge is elicited by means of concept maps.

The first difference might be in the articulation of basic science concepts. As medical expertise develops, basic science knowledge gradually becomes encapsulated by clinical concepts as a consequence of clinical exposure. This is evidenced by studies showing that experienced clinicians mention fewer basic science concepts when they diagnose patient cases (Van de Wiel et al. 2000) than residents that have not yet ‘wrapped up’ their basic science knowledge in clinical knowledge to the same extent (Boshuizen and Schmidt 2000; Van de Wiel et al. 2000). It is during the residency that basic science knowledge gradually becomes intertwined with situated, clinical knowledge. Hence, residents might more easily articulate basic science concepts in concept maps than experienced clinicians.

Another difference between concept maps of experienced clinicians and resident concept maps might be the use of clinical concepts to organize the concept map. When experienced clinicians recall patient cases, they tend to use clinical summaries or higher order clinical concepts whereas 6th year students, for example, articulate many more detailed concepts of a basic scientific nature (Boshuizen and Schmidt 2000; Van de Wiel et al. 2000). Experienced clinicians use these higher order clinical concepts to organize the information they give about a patient case. Hence, we hypothesize that the organization of concept maps of experienced clinicians relies more on clinical concepts that encapsulate basic science concepts, whereas the expectation is that residents will more frequently use basic science concepts.

The third characteristic that might distinguish expertise levels emanates from the cohesion that pervades expert behaviour. By means of a knowledge network with a high density of interrelations between concepts (Eva et al. 2002a; Feltovich et al. 1993; Genberg 1992), experts bring together symptoms and complaints into a diagnosis or underlying basic science mechanism (Eva et al. 2002a; Feltovich et al. 1993). They are inclined to connect data from a holistic viewpoint (Eva et al. 2002a). We hypothesize that this holistic viewpoint results in more links between clinical and basic science concepts in concept maps of experienced clinicians compared to resident concept maps.

There is educational utility in exploring how groups consisting of experienced clinicians and basic scientists differ from those comprised of residents and basic scientists-in-training in their articulation of the integration of clinical and basic sciences. Insights into these differences could enable us to make decisions about the expertise level of the constructors of preconstructed concept maps and the role of residents in curriculum development when we are faced with the task of developing integrated course content. So far, there have been no studies focusing on groups with different expertise levels that articulate integration of clinical and basic science concepts. Studies that map out differences in complexity of concept maps aim to elicit knowledge structures of individuals (McGaghie et al. 2000; Rendas et al. 2006; West et al. 2000). However, concept maps for integrated curricula, like any other means to improve integration (Harden 2000), seem to be preferably constructed by *groups* of constructors. These maps reflect the negotiations of the participants about what they consider to be relevant concepts and relations.

We aimed to investigate the consistent variations between concept maps constructed by groups consisting of experienced clinicians and basic scientists, and those constructed by groups of residents and basic scientists-in-training. In our analysis we focused on the articulation of integration of clinical and basic sciences and expected to find differences regarding the number of basic science concepts, the number of links between clinical and basic science concepts, and the way in which the integration of clinical and basic science concepts is organized. Moreover, concept mapping studies have revealed increasing complexity of organization reflecting individual constructors’ growing expertise (McGaghie et al. 2000; West et al. 2000). This increasing complexity might also be found if a concept map is constructed by a group rather than by an individual constructor.

## Methods

### Context and participants

We conducted a study at Leiden University Medical Centre that aims to offer an integrated bachelor curriculum. Seven groups of experienced clinicians and basic scientists, i.e. specialists with a basic science specialization such as anatomy, pathology, immunology, and who do not have patient encounters on a frequent basis (’experienced groups’), and seven groups of residents and basic scientists-in-training (‘resident groups’) were asked to articulate their knowledge about a clinical problem by means of a concept map. Each group consisted of three participants and the experienced groups and the resident groups had an equivalent disciplinary composition (see Table 1 for the composition of the groups). The multidisciplinary character of the groups was expected to contribute to the articulation of their knowledge due to the information gap between the participants. The participants in the experienced groups had worked as certified specialists in their domain for at least 5 years (most of them for more than 20 years), and next to their patient care and research, were all highly involved in teaching programmes, often both as teacher and instructional designer. The participants in the resident groups were doing their residency or were following their specialization programme as basic scientists. For practical reasons, there were no requirements regarding the phase of the programme they were doing. Just a few of them had some experience in teaching, i.e. coaching clerks in clinical programmes. None of the participants were familiar with concept mapping; only a few participants of the experienced groups had worked with the schemes as proposed by Mandin et al. (1997), i.e. concise clinical reasoning schematizations.

**Table 1:** Composition of both experienced and resident groups. All groups consisted of 3 participants. Disciplines indicated with an * are viewed as basic sciences

Concept map	Discipline of each participant
Blood in faeces	GP
	Pathology*
	Surgery
Chronic abdominal pain	Radiology/anatomy*
	Gynaecology
	Internal diseases
Cough	Infectious diseases
	Immunology*
	Lung diseases
Diarrhoea	Anatomy*
	Gastro-internal diseases
	Infectious diseases
Diarrhoea	Gastro-internal diseases
	Microbiology*
	Surgery
Painful joints	Immunology*
	Rheumatology
	Surgery
Proteinuria	Gynaecology
	Pathology*
	Nephrology

### Materials and procedure

Each group was asked to articulate their knowledge by constructing collaboratively a concept map about a clinical problem. The clinical problems emanated from internal medicine but were also relevant for other clinical disciplines: coughing, diarrhoea (2x), proteinuria, blood in faeces, chronic abdominal pain and articular pain. Diarrhoea was schematized twice but the disciplinary composition of the groups differed.

**Fig. 1:**
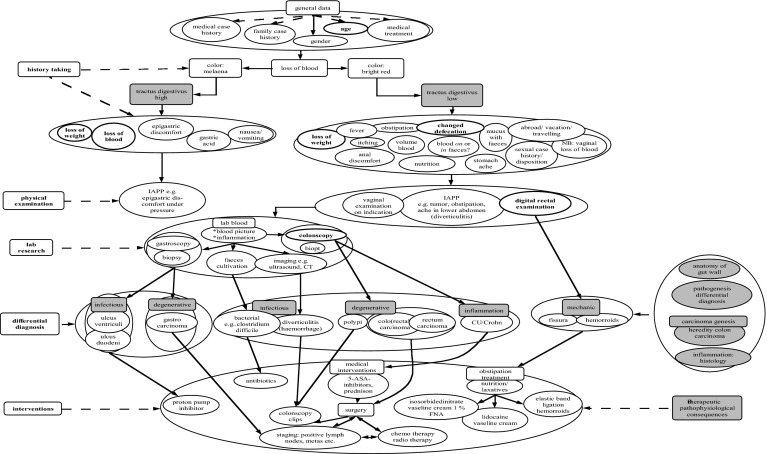
Concept map about blood in faeces, constructed by a resident group: a GP, a surgeon and a pathologist. Basic science concepts are *grey colored*. Umbrella concepts are *rectangular*

**Fig. 2:**
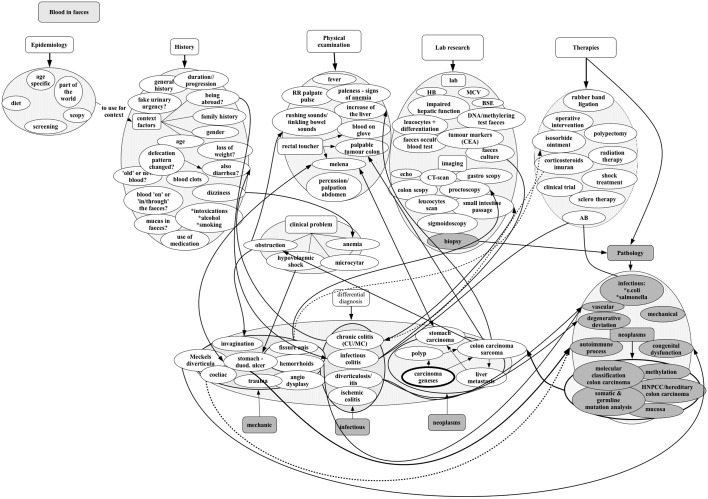
Concept map about blood in faeces, constructed by an experienced group: a GP, a surgeon and a pathologist. Basic science concepts are *grey colored*. Umbrella concepts are *rectangular*

The concept maps were created during two 2-h sessions. In the first session, one of the researchers (SV) explained the aim of the concept mapping sessions, i.e. to make explicit integrated clinical and basic science knowledge that would be helpful to understand the clinical problem. She guided the participants through concept mapping instructions designed to support visualization of integration: the constructors were instructed to contribute concepts that were particularly relevant from the perspective of their own discipline, and to explore links between clinical and basic science concepts. During each phase of the concept mapping session the researcher reminded the participants to keep their own disciplinary perspective and to check how the clinical and basic science concepts they added to the map could be linked. She naturally did not comment on the content, because this study focused on what the groups decided should be incorporated. However, in each phase, she encouraged the groups to go on until consensus had been achieved about what to incorporate in the concept map. They then had to explain two complex patient cases using the concept map in order to check whether the map was comprehensive. The researcher checked whether all participants considered the concept map sufficiently informative. Concept maps were created using post-it notes and large paper sheets, and were digitized using *Inspiration@*, a software tool by the researcher (SV) to create concept maps after the first session. The second session was spent on checking the digitized version and on elaborating and fine-tuning the concept map (Vink et al. 2015). Figures 1 and 2 in Appendix show concept maps on blood in faeces constructed by a resident group and an experienced group: a GP who contributed general data, history, and obstipation treatment, a surgeon whose contribution focused on degenerative aspects, history and physical examination, while a pathologist contributed degenerative and inflammation aspects. The Institutional Board of Leiden University Medical Centre, where the concept maps were constructed, provided ethical approval for the study.

### Data analysis

A framework of features was developed to describe the concept maps. The features enabled us to quantify differences between the concept maps, in particular on the issue of integration. We distinguished concepts (clinical concepts and basic science concepts); organization (umbrella concepts and clinical-problem-specific umbrella concepts); and integration (clusters, hierarchies and links) (Vink et al. 2015). These features were distinguished after several rounds of analyses, grounded in the data as presented in the concept maps.

*Concepts*: in Fig. 1 (see Appendix), the clinical concepts are those under general data, history-taking and physical examination, lab research, diagnosis and interventions. The basic science concepts are indicated by grey nodes. Resident concept maps were expected to cover relatively more basic science concepts than concept maps of the experienced groups. Therefore, the proportion of both were computed.

*Organization* of the concept maps was measured by umbrella concepts. All rectangular concepts in Fig. 1 (see Appendix) are interpreted as umbrella concepts. We distinguished general umbrella concepts that are applicable to every clinical problem (in Fig. 1 (see Appendix), for example, history-taking, physical examination, lab research and differential diagnosis) and umbrella concepts that are specific to the clinical problem of the concept map such as tractus digestivus high, infectious, colour: bright red in Fig. 1 (see Appendix). Umbrella concepts that are clinical-problem-specific have been shown to correlate with level of expertise (Rendas et al. 2006; West et al. 2000).

*Integration* is described by means of clusters, hierarchies and links. Integrated clusters are groups of at least one clinical and one basic science concept placed (either hierarchically or non-hierarchically) close to each other. See Fig. 1 in Appendix: tractus digestivus high and the five concepts—loss of weight, loss of blood, epigastric discomfort, gastric acid and nausea/vomiting—are interpreted as a cluster because it contains both basic science concepts and clinical concepts. In a hierarchy, there is always an umbrella concept that is explained by one or more other concepts. Two types of integrated hierarchies were distinguished and counted to measure differences in concept maps of resident and experienced groups: clinical concepts encapsulating basic science concepts (Fig. 1 in Appendix, loss of blood encapsulates tractus digestivus high and tractus digestivus low) and basic science concepts that subsume clinical concepts (Fig. 1 in Appendix: infectious subsuming ulcus ventriculi and ulcus duodeni). Links between clinical and basic science concepts were counted, mapping out links between basic science concepts and (1) history and physical examination, (2) lab research, (3) diagnosis, and (4) interventions in order to gauge the role of integration in the different phases of clinical reasoning. An example in Fig. 1 (see Appendix) is the link between the concepts “digital rectal examination” and “mechanic”. Links could articulate either hierarchical or non-hierarchical relations. The experienced groups are expected to generate more links between clinical and basic science concepts than the residents as a reflection of a more holistic view (Feltovich et al. 1993).

Two researchers analysed the concept maps and decided what should be considered as a concept or a cluster. Some concepts could be interpreted as either one or two concepts (e.g. the basic science concepts ‘inflammation: histology’ in Fig. 1, see Appendix). Consensus was achieved about the interpretations, so that discrepancies that were irrelevant to our aim would not blur the analysis. Subsequently, the concept maps were analysed along the feature framework described above by two researchers. Part of the data was analysed by another researcher in order to check the interrater reliability of the analysis. Cohen’s kappa ranged from .87 to 1.00. The mean Cohen’s kappa was .95. Two researchers were blinded as to whether the concept map was constructed by an experienced group or by a resident group. Differences between concept maps of experienced and resident groups were analysed by independent *t* tests.

## Results

Table 2 gives an overview of the extent and type of articulation of integration when clinicians and basic scientists are guided by concept mapping instructions. It focusses on the differences between the concept maps constructed by the experienced groups and those constructed by the residents groups as measured by concepts, organization and integration. The resident concepts maps tended to be more elaborate, regarding the number of both concepts and relations. The number of clinical and basic science concepts used by the two expertise levels differed, but not significantly. The same was true for the proportion of basic science concepts and clinical concepts. Standard deviations were relatively high.

**Table 2:** Means and standard deviations for the features that describe the concept maps constructed by resident and experienced groups and their differences measured with independent *t* tests

	Resident concept maps N = 7		Expert concept maps N = 7		
	Mean	SD	Mean	SD	*t*
Concepts					
Clinical concepts	85.6 (75 %)	18.3	70.7 (79 %)	19.7	1.46
Basic science concepts	28.3 (25 %)	21.3	19.1 (21 %)	7.6	1.07
Organization					
Total umbrella concepts	24.9	6.7	14.6	3.6	3.56**
General umbrella concepts	7.0	3.8	5.3	3.8	.84
Clinical-problem-specific umbrella concepts	17.9	6.7	9.6	3.3	2.95*
Integration					
Clusters of clinical and basic science concepts	12.0	3.2	2.3	3.7	5.26**
Hierarchies					
Clinical concepts encapsulating basic science concepts	0.7	1.5	2.9	3.9	−1.36
Basic science concepts subsuming clinical concepts	9.4	3.9	2.6	4.4	3.10**
Links between clinical and basic science concepts:	24.0	6.0	6.9	4.8	5.88**
History, physical examination + basic science	6.3	3.5	0.9	1.2	3.83**
Lab + basic science	4.3	2.9	0.3	0.5	3.64*
Diagnosis + basic science	11.1	5.7	5.1	4.2	2.24*
Interventions + basic science	2.3	2.5	0.6	0.8	1.73

* *p* < 0.05

** *p* < 0.01

The organization of the concept maps of experienced clinicians and basic scientists differed significantly from that in the maps by the groups on resident level. The concept maps at resident level encompassed significantly more umbrella concepts, that is concepts that subsume other concepts [24.9 ± 6.9 vs 14.6 ± 3.6; *t*(12) = 3.56, *p* = .006] due to significantly more clinical-problem-specific umbrella concepts than the concept maps at experienced level [17.9 ± 6.7 vs 9.6 ± 3.3, *t*(12) = 2.95, *p* = .017].

To visualize integration, the resident groups gave basic science concepts a prominent role, leading to significant differences with the concept maps of the experienced groups. They clustered clinical and basic science concepts more frequently than experienced groups [12.0 ± 3.2 vs 2.3 ± 3.7, *t*((12) = 5.26, *p* = .000] and structured the hierarchies significantly more along basic science concepts that subsumed clinical concepts than experienced groups did [9.4 ± 3.9 vs 2.6 ± 4.4, *t*(12) = 3.10, *p* = .009]. Although not statistically significant due to a fairly high spread, experienced groups employed clinical concepts as an organizational device more frequently [2.9 ± 3.9 vs 0.7 ± 1.5].

Contrary to our expectations, links between clinical and basic science concepts were used significantly more frequently by the resident groups [24.0 ± 6.0 vs 6.9 ± 4.8, *t*(12) = 5.88, *p* = 000]. Additionally, we investigated which type of clinical concepts were preferably linked with basic science concepts: history and physical examination, lab research, diagnosis or interventions, to get an impression of the role of basic science concepts in the different phases of the clinical reasoning process. All these four combinations were articulated more by resident groups with significant differences between concept maps of the two expertise levels when basic science concepts were linked to history and physical examination [6.3 ± 3.5 vs 0.9 ± 1.2, *t*(12) = 3.83, *p* = .006], to lab [4.3 ± 2.9 vs 0.3 ± 0.5, *t*(12) = 3.64, *p* = .010], and to diagnosis [11.1 ± 5.7 vs 5.1 ± 4.2, *t*(12) = 2.24, *p* = .047], again underlining the role of basic science in the concept maps at resident level in the clinical reasoning process. Note that all these significant differences had moderate to high effect sizes, calculated by Cohen’s *d* ranging .51–.84 (not shown).

The quantitative differences in organization and integration between the concept maps of the experienced and resident groups became apparent in their appearance. In some concept maps of the resident groups, a concept-map-like appearance, that is concepts structured in a hierarchical way, was combined with a matrix in which the headings of the columns were basic science categories. One of the resident groups described this as ‘the magical ruler’ that helps us to understand the clinical concepts. In the concept maps of the experienced groups, mainly clusters were linked (instead of concepts), resulting in a relatively low number of links. This strengthens the image of the ‘expert’ concept maps as resembling archipelagos—as one participant described the process of organizing the concept maps: ‘let’s make islands, so that we can do some island hopping’. This metaphor adequately describes the organization of most concept maps of the experienced groups: clusters of concepts organized by the phases in the diagnostic process: history taking and physical examination, lab research, diagnosis and interventions.

## Discussion

This study demonstrates that groups of experienced clinicians and basic scientists, on one hand, and residents and basic scientists-in-training, on the other hand, differ in both the extent and how they articulate integration. These differences have particular relevance when it comes to constructing curricula aimed at the integration of clinical and basic sciences. Hence, it provides support for further investigation of the role that preconstructed resident concept maps could play for the development of integrated curricula and as a scaffold for student learning. Educational programmes rely on knowledge that can be made explicit and discussed in the classroom (Eraut 2000). Because the resident concept maps articulated decidedly more integration of clinical and basic sciences than those of the experienced groups, their role in helping to determine the course content of integrated course modules and in guiding students through the literature and analysis of patient cases seems to be worth exploring.

How did integration become apparent in the concept maps of the resident groups in contrast to the concept maps of the experienced groups? We hypothesized three variations between the concepts maps at the two expertise levels. First, the hypothesis that groups of residents and basic scientists-in-training would articulate more basic science concepts than groups of experienced clinicians and basic scientists could not be completely supported. Overall, their concept maps contained more basic science concepts but the difference was not significant, due to the fair variation with respect to the number of concepts they covered. Second, based on findings in think-aloud protocols in studies on knowledge organization (Boshuizen and Schmidt 2000; Van de Wiel et al. 2000), the resident groups were expected to use basic science concepts for the organization of the concepts to a higher extent whereas the experienced groups were expected to rely more on clinical concepts that encapsulate basic science concepts. The resident groups did indeed mainly organize their maps through the organizational device of basic science categories and they did this significantly more than the experienced groups. Experienced clinicians and basic scientists used both devices to organize and integrate the clinical and basic science concepts in their map. Thus the organization of concept maps was partly consistent with our expectations: the residents organised their concept maps as we expected, the experienced groups showed only a slight preference for clinical concepts that encapsulated basic science concepts. Such a flexible use of two devices to organize their concept maps might develop with the expertise level of the group of constructors, a process that is equivalent to the growing flexibility in using reasoning strategies and knowledge of individual clinicians (Eva et al. 2002b). However, overall, the experienced groups were reluctant to organize their concept maps along hierarchical devices. Their organization followed the phases of clinical reasoning along which the concepts were clustered. This clustering along the clinical reasoning process might be due to the highly developed procedural knowledge of experienced clinicians as described in cognitive literature, enabling them to quickly process clinical information and hypothesize a few diagnoses (Feltovich et al. 2006; Schraw 2006). Encapsulation of basic science knowledge under clinical diagnostic reasoning categories (Boshuizen and Schmidt 2000; Van de Wiel et al. 2000) might thus be seen as the development of procedural knowledge. Third, the hypothesis that experienced groups would articulate more links between clinical and basic science concepts than resident groups due to their holistic approach to solving problems (Feltovich et al. 1993, 2006) turned out not to be the case. Resident groups proved to be more ‘expressive’ than experienced groups in the use of links to articulate integration of clinical and basic science concepts. Both groups preferred to connect basic science concepts with diagnoses, but resident groups also articulated links between basic science concepts and concepts used earlier in the clinical reasoning process: history, physical examination and lab research. The reluctance of the experienced groups to link basic science concepts to concepts regarding history, physical examination and lab research may be explained by the prevalence of procedural knowledge during clinical reasoning (Feltovich et al. 2006).

As a knowledge elicitation technique, concept mapping is supposed to unveil the knowledge of both individuals and groups (Hoffman and Lintern 2006; Novak 2002). However, the contribution of group dynamics has not been taken into account in previous concept mapping studies. In our study, group dynamics were supposed to affect the concept maps on both expertise levels equally, because the disciplinary composition of the groups were the same. However, these group dynamics may have contributed to the differences between the articulation of integration in the ‘resident’ or ‘experienced’ concept maps. Although the multidisciplinary composition of the groups was assumed to create an information gap that would affect the need to share and therefore articulate knowledge, the experienced clinicians and basic scientists might have been aware of the fact that they all were indeed highly experienced or even experts, evoking more concise explanations. The social dimension might have influenced the process of concept mapping: experts among experts might tend to explicate less assuming that their interlocutors could rely on the same knowledge base. This might also explain why the experienced groups left hierarchical relations unarticulated, linked clusters instead of concepts, and followed the diagnostic reasoning process by using relatively many general umbrella concepts like history and physical examination. In further research, the social dimension of constructing concept maps could be manipulated by groups consisting of both residents and experienced clinicians, possibly resulting in articulation of relations. Furthermore, the experienced clinicians and basic scientists, often involved in medical teaching, aimed to reduce the complexity of the concept map. This fine-tuning of the concept map for educational purposes—scaffold for preclinical and clinical students—might have encouraged them to leave relations implicit which in other studies might have been articulated (Kassab and Hussain 2010; Rendas et al. 2006). The resident groups seemed to bother less about the complexity of the concept maps but were more concerned about the articulation of causal basic science concepts and the organization of the map, which was reflected by their use of clinical-problem-specific umbrella concepts. This is at odds with previous studies on individual concept mapping that show that the higher the expertise level, the higher the degree of organization (McGaghie et al. 2000; Rendas et al. 2006).

The expressiveness of the resident groups regarding integration reminds us of the intermediate effect described within the context of encapsulation theory, which addresses the knowledge structures of individuals (Van de Wiel et al. 2000): intermediates tend to mention not only more basic science mechanisms but also more clinical data than experts (Van de Wiel et al. 2000). Although the resident groups in our study are surely more knowledgeable than the intermediate levels that are referred to in these studies, they showed the same tendency when they constructed concept maps in groups. In order to explore whether there is indeed an intermediate effect in concept maps that are constructed in groups, this study should be duplicated with students who are expected to construct less elaborate concept maps.

### Educational implications

Evidently, we need to be cautious with our conclusions because of the limited number of concept maps that we were able to include in this study. However, the consistent explicitness of concept maps constructed by the resident groups might make them more suitable for determining the course content of integrated curricula. For the purpose of developing integrated modules it would therefore be worthwhile to involve residents, in order to determine which basic science concepts and which of their relations with clinical concepts should be incorporated. Scrutinizing these modules might reveal whether they entail more explicit attention to the relations between clinical and basic sciences than modules designed by experienced clinicians and basic scientists, as they usually are.

Whether students would experience resident preconstructed concept maps as a better format for supporting their knowledge acquisition requires further investigation. The increase in complexity due to the articulation of integration may be counterproductive for student learning. However, the explicit articulation of integration might support students as they attempt to relate clinical and basic science knowledge. Furthermore, the articulated relations between history, physical examination and lab research on the one hand and basic science concepts on the other hand might be helpful for medical students, because these relations often remain underexposed in medical curricula (Kulasegaram et al. 2013; Prince and Boshuizen 2004). In teaching there is a tendency to simplify and reduce information, even if the learners are residents (Cutrer et al. 2011). Our findings question whether this is necessary and fruitful. The use of concept maps as an advance organizer, with the particular aim of students’ learning to integrate clinical and basic sciences, is still in its infancy. Preconstructed concept maps and concept maps which should be partly completed by students (Rendas et al. 2006) might complement approaches in which students construct concept maps by themselves. Contrasting these different approaches might help our understanding of students’ learning processes regarding the integration of clinical and basic sciences.

Educational programmes aim to support medical students in achieving knowledge organization fit for clinical practice, by helping them to organize their knowledge around clinical concepts (Prince and Boshuizen 2004). Expert concept maps are in congruence with the knowledge organization that should be acquired in the long run. However, organizing clinical concepts along basic science concepts, as the resident groups mainly did, might be a necessary developmental phase in the building of a sound integrated medical knowledge base and such concept maps might therefore complement a clinically oriented medical programme by bridging the gap between expert knowledge organization and student knowledge organization.
